# Impact of oral melatonin on critically ill adult patients with ICU sleep deprivation: study protocol for a randomized controlled trial

**DOI:** 10.1186/1745-6215-15-327

**Published:** 2014-08-18

**Authors:** Huawei Huang, Li Jiang, Ling Shen, Guobin Zhang, Bo Zhu, Jiajia Cheng, Xiuming Xi

**Affiliations:** Department of Critical Care Medicine, Fuxing Hospital, Capital Medical University, 20A Fu Xing Men Wai Da Jie, Xicheng District Beijing, 100038 China; Department of Otorhinolaryngology, Fuzhou Children’s Hospital of Fujian Province, Teaching Hospital of Fujian Medical University, Ba Yi Qi Zhong Road, Gulou District, Fuzhou Fujian, 350005 China; Department of Neurosurgery, Beijing Tiantan Hospital, Capital Medical University, Tiantan Xili 6, Chongwen District Beijing, 100050 China

**Keywords:** Melatonin, Sleep deprivation, Anxiety, Polysomnography

## Abstract

**Background:**

Sleep deprivation is common in critically ill patients in intensive care units (ICU). It can result in delirium, difficulty weaning, repeated nosocomial infections, prolonged ICU length of stay and increased ICU mortality. Melatonin, a physiological sleep regulator, is well known to benefit sleep quality in certain people, but evidence for the effectiveness in ICU sleep disturbance is limited.

**Methods/Design:**

This study has a prospective, randomized, double-blind, controlled, parallel-group design. Eligible patients are randomly assigned to one of the two treatment study groups, labelled the ‘melatonin group’ or the ‘placebo group’. A dose of 3 mg of oral melatonin or placebo is administered at 9:00 pm on four consecutive days. Earplugs and eye masks are made available to every participant. We plan to enrol 198 patients. The primary outcome is the objective sleep quality measured by the 24-hour polysomnography. The secondary outcomes are the subjective sleep quality assessed by the Richards Campbell Sleep Questionnaire, the anxiety level evaluated by the Visual Analogue Scale-Anxiety, the number of delirium-free days in 8 and 28 days, the number of ventilation-free days in 28 days, the number of antibiotic-free days, ICU length of stay, the overall ICU mortality in 28 days and the incidence and severity of the side effects of melatonin in ICU patients. Additionally, the body stress levels, oxidative stress levels and inflammation levels are obtained via measuring the plasma melatonin, cortisone, norepinephrine, malonaldehyde(MDA), superoxide dismutase(SOD), interleukin-6 (IL-6) and interleukin-8 (IL-8)concentrations.

**Discussion:**

The proposed study will be the first randomized controlled study to use the polysomnography, which is the gold standard of assessing sleep quality, to evaluate the effect of melatonin on the sleep quality and circadian rhythms of ICU patients. The results may recommend a new treatment for ICU patients with sleep deprivation that is safe, effective and easily implementable in daily practice.

**Trial registration:**

This study was registered with ClinicalTrials (NCT; registration number: ChiCTR-TRC-14004319) on 4 March 2013.

## Background

Sleep deprivation is a major concern in critically ill patients in intensive care units (ICU). Several studies have shown that poor sleep quality and the inability to sleep are the second largest stressors and rank among the top three major sources of anxiety during ICU stays [1–3]. Sleep for ICU patients is characterised by frequent disruptions, loss of circadian rhythms and a paucity of time spent in restorative sleep stages. Typical findings described by polysomnography (PSG), the gold standard of assessing sleep quality, include increased latency, a higher proportion of non-rapid eye movement (NREM) sleep stage 1 and 2 (or light sleep), and reduced restorative slow wave (SW) and rapid eye movement (REM) sleep, largely because of frequent waking. Although ICU patients may experience normal or near normal total sleep time (TST), approximately 50% of this sleep occurs during the daytime [4, 5]. In a recent observational study by Elliot *et al.* a 24-hour PSG was used to evaluate the sleep quality in ICU patients [5]. They found that despite improvements in ICU design, technology and healthcare personnel training, there has been no improvement in the ICU sleep problem [5–7]. It has been found that there are many extrinsic and intrinsic factors of sleep deprivation in the ICU setting including noise, light, nursing procedures, the presence of existing diseases, inflammatory mediators, anxiety, pain, sedative and opioid medications and mechanical ventilator setting [8–12]. Furthermore, the occurrence of ICU sleep deprivation is associated with detrimental outcomes, including delirium, difficulty weaning, increased nosocomial infections, prolonged ICU length of stay (LOS) and increased ICU mortality [13].

Conversely, despite the poor sleep quality that disturbs almost all ICU patients, clinicians remain reluctant to administer traditional sedative-hypnotic drugs in patients with sleep disorders. The major concerns are the side effects of these drugs, mainly that they destroy the structure of sleep, reduce the clinician’s ability to monitor the level of consciousness, induce respiratory depression and lower blood pressure [14].

Sleep goals for ICU patients are to get enough sleep, reset the disordered circadian rhythms, adjust the abnormal sleep structure, reduce sleep interruption, overcome fatigue and anxiety, facilitate nursing care and treat disease. An ideal therapy for improving sleep in the ICU should be economical, feasible, rapid in onset and offset and without local and systemic adverse effects. At present, there is no effective treatment in use to improve ICU sleep that covers all of these ideal properties. Current studies are mainly focused on non-drug treatments such as earplugs and/or eye masks [10, 15] and imagery and relaxation [16] *.* These treatments are relatively safe but do not guarantee efficacy. Among the studies, the clinical research on earplugs and/or eye masks has some maturity [15]. Some domestic and international experts and scholars have recommended that ICUs incorporate earplugs and/or eye masks into routine nursing care [17]. However, Bourne *et al*. [18] and Gabor *et al*. [7] showed that environmental factors were responsible for a fraction of arousals and awakenings, and Perras *et al*. [19] indicated that the physiological regulation of melatonin secretion by darkness and light was abolished in severely ill patients in the ICU. Therefore, treatments based on environmental factors might have limited effects. Recently melatonin, a physiological sleep aid, has gained interest among ICU scholars.

Melatonin (N-acetyl-methoxytryptamine) is a neurohormone mainly secreted by the pineal gland. Light signals play the most important role in the synthesis and secretion of melatonin in organisms. Thus, the environmental cues that regulate an organism’s biological clock are predominantly the daily alternation of light and darkness acting via the retina and retina-hypothalamic pathways directly on the suprachiasmatic nuclei (SCN). Melatonin secretion increases directly with the length of darkness. Increased light intensity decreases the quantity of endogenous melatonin produced and shifts the pattern of release throughout the circadian clock. Endogenous melatonin is released at night, beginning at approximately 9:00 pm with a peak release at between 2:00 and 4:00. Melatonin release is typically inhibited between 7:00 and 9:00, coinciding with the peak of endogenous cortisol [20]. This secretion pattern makes the physiological activities in the human body, such as the sleep-wake cycle, synchronised with the circadian rhythm. Thus, melatonin is a good sleep aid. In addition, current *in vitro* and *in vivo* experiments suggest that melatonin might act as a mood stabilizer, relieve stress, act as an anti-oxidation and anti-inflammation agent, suppress pathogens and protect the functioning of multiple organs [20], which are undoubtedly helpful to the recovery of ICU patients, and thereby might improve sleep.

Prolonged-release melatonin (Circadin), an oral medication to regulate physiological sleep and the circadian rhythm designed to mimic the endogenous pattern of melatonin production, is licensed for the treatment of primary insomnia in patients aged 55 years and over. It results in significant and clinically meaningful improvements in sleep quality, morning alertness, sleep onset latency and quality of life, without withdrawal symptoms upon discontinuation [21]. Recently, extensive clinical trials also noted that melatonin could be beneficial in different populations with sleep disorders. Firstly, melatonin might be effective for insomnia and daytime sleepiness caused by time zone changes [22] and work shifts [23] that induce the malfunctioning of biological clocks. This is because melatonin may maintain the synchronisation in situations where the circadian rhythms are jeopardized and resynchronize after a period of free-run release. Secondly, melatonin might improve the sleep quality of non-ICU critically ill patients with dialysis [24], moderate to severe COPD [25] and asthma [26]. In addition, the available clinical data shows that perioperative use of melatonin is effective in reducing preoperative anxiety [27] and plays a role in the prevention of postoperative delirium [28], as well as possessing certain analgesic qualities, and may reduce concomitant opioid use in the postoperative period with a corresponding reduction in opioid-associated side effects [29].

Melatonin has been given safely to humans in doses of 1 to 15 mg. Although treatment results in plasma levels up to 100 times the normal peak night concentration approximately 1 hour after ingestion, it has a wide safety margin [30]. In a meta-analysis, Buscemi *et al*. concluded that melatonin is safe for short-term use [31]. They found that the most common side effects of melatonin use were headache, dizziness, nausea and drowsiness [31]. Most importantly, although melatonin has hypnotic, sedative and analgesic properties, it has few respiratory and hemodynamic effects.

The interest in melatonin as a potential therapeutic or prophylactic agent in the management of sleep disturbance in the ICU derives from the demonstrated low plasma concentrations and altered secretion patterns of melatonin in critically ill patients. Shilo *et al*. studied the day secretions of melatonin in a group of ICU patients compared to a group of patients in ordinary medical wards. They found that the nocturnal peak of melatonin was missing in most ICU patients [32]. Mundigler *et al*. described a disturbed pattern of circadian secretion of melatonin in ICU patients with sepsis (16 out of 17 patients) but a preserved circadian rhythm in ICU patients who did not have sepsis (six out of seven patients) [33]. Olofsson *et al*. found that the circadian rhythm of melatonin secretion was abolished in mechanically ventilated patients in the ICU [34]. Perras *et al*. suggested that the nocturnal melatonin concentrations in ICU patients were negatively correlated with illness severity [19]. In addition, various drugs commonly used in the ICU have been reported to alter melatonin secretion and to decrease the plasma levels of melatonin [35] including benzodiazepines, non-steroidal anti-inflammatory drugs (NSAIDs), corticosteroids and beta-blockers. Therefore, low melatonin levels, poor sleep quality and illness have a reciprocal causation interaction and form a vicious circle. The supplementation of exogenous melatonin to remodel the melatonin level in the human body that approaches the physiological state might be one of most effective strategies for improving sleep.

Both melatonin and cortisol are biological markers of the circadian rhythm. Some previous studies have shown that there is a hypo-secretion of melatonin and an overall high cortisol excretion in most patients in the ICU. Cortisol is an important stress hormone that would be invoked by the noise, light and other stressors in the ICU and leads to anxiety and sleep disturbance. It is known that melatonin can reduce the adrenocortical response to stress and down-regulate the synthesis and release of cortisol. Moreover, in addition to reducing the stress, the role of melatonin as an antioxidant and anti-inflammatory agent or part of sepsis treatment is widely discussed. Thus, administration of melatonin might significantly benefit ICU patients.

Recently, Mistraletti *et al*. studied the pharmacokinetics of melatonin given orally to ICU patients and found a good oral bioavailability of the drug [36]. Additionally, several studies suggest it can take up to three days to achieve the desired effect of melatonin on sleep quality [13, 18, 30, 37]. Until now, there have been only three studies investigating the influence of melatonin treatment on sleep quality in critically ill patients. Shilo *et al*. [37] and Bourne *et al*. [18] found that melatonin improved sleep quality and sleep length in critically ill patients in the ICU, however Ibrahim *et al*. [30] found a negative result in their study. There are some inconsistencies regarding the inclusion criteria, the drug included, the sound and light control and the monitoring method in these three studies, specifically as follows:(1) Ibrahim *et al*.’s study included patients who had unlimited use of sedatives and analgesics that might affect serum melatonin levels; (2) there was no uniformity for the control of patient exposure to noise and light among the three studies, which may have a more powerful effect on observed sleep than even the pharmacological levels of melatonin achieved; and, most significantly, (3) they did not use the PSG (the gold standard for assessing sleep) to evaluate sleep quality and ignored the importance of monitoring the all-day sleep, likely because it is very difficult to perform 24-hour PSG in severely ill patients in the ICU.

The aim of the present work is to evaluate the efficacy and safety of melatonin for ICU sleep deprivation. Our hypothesis is that melatonin will improve the sleep quality in ICU patients. The present article proposes a protocol for a clinical study consisting of a double-blind, randomized, placebo-controlled trial with melatonin in adult critically ill patients with ICU sleep deprivation. The present report will follow the guidelines expressed by the Consolidated Standards of Reporting Trials (CONSORT).

## Methods/Design

### Study design and outcomes

The present study is a prospective, single-centre, randomized, double-blind, placebo-controlled, two-arm trial in patients with sleep deprivation in the ICU. All eligible patients in the ICU will be 1:1 randomized to the treatment intervention with melatonin or placebo. The objective of the trial is to evaluate the efficacy and safety of melatonin for ICU sleep deprivation. The definitions of the variables in the objectives are reported in Table 1.

**Table 1:** **Definition of study objectives**

Objective	Definition
Objective sleep quality	Total sleep time, the length of sleep at night (9:00 pm - 7:00 am) and during the day (7:00 am - 9:00 pm), sleep architecture (NREM stage 1 and 2, SWS, REM), and sleep disruption (the number of arousals and duration of sleep), which will be monitored for one 24-hour period using a portable PSG device.
Subjective sleep quality	The subjective feelings of nightly sleep status of the patient, including sleep depth, wake time after sleep onset, number of awakenings after sleep onset, latency to sleep onset, and sleep quality. It will be evaluated using the RCSQ [38, 39].
Anxiety level	ICU anxiety is defined as a state marked by apprehension, agitation, increased motor activity, number of arousals, and fearful withdrawal during the ICU stay [40]. It will be assessed using the VAS-A.
Delirium-free days in 8 and 28 days	Number of days that the patient is not delirious over 8 and 28 days starting from the day of inclusion. Patients are diagnosed as delirious when they have at least one positive CAM-ICU screening during their ICU stay. A delirium-free day is defined as a negative CAM-ICU screening during that day. In case a delirious patient is discharged from the ICU, a delirium-free day is defined as a delirium observation scale score of less than 3 during a complete day [41].
Ventilator-free days in 28 days	Time in days that the patient is not on a mechanical ventilator. If the patient is ventilated mechanically, including invasive and non-invasive ventilation several times during one ICU admission, then the non-ventilator times are added. Ventilator-free days (in 28 days) will be calculated.
Antibiotic-free days in 8 and 28 days	Number of days that the patient does not require any antibiotics at 8 and 28 days from randomization will be calculated.
ICU length of stay	Duration of admission to the ICU.
Overall ICU mortality at 28 days	Survival time will be assessed. Patients will be classified as either alive at study day 28 or dead at study day 28. Differences between the two strategies in mortality rates will be evaluated using the assumption of asymptotic normality. Estimates of relative risks and odds ratios and the corresponding 95% confidence intervals will be presented.
Side effects	Headache, dizziness, nausea, and drowsiness, determined daily by physical examination by the intensivist, and withdrawal symptoms upon discontinuation evaluated after the drugs are stopped.

NREM, None rapid eye movement; SWS, Slow wave sleep; REM, Rapid eye movement; PSG, Polysomnography; RCSQ, Richards Campbell sleep questionnaire; VAS-A, Visual analogue scale-anxiety; CAM-ICU, The confusion assessment method for the intensive care unit.

The primary outcome will be to determine the effect of melatonin administration on the 24-hour sleep-wake cycle and subjective sleep quality, including night/day sleep time, total sleep time(TST), percentages of NREM stage 1/2, slow wave sleep(SWS) and REM, the incidence of arousals per hour, the duration of sleep without waking and the number of sleep periods.

The secondary outcomes will be: (a) objective sleep quality as assessed by the Richards Campbell Sleep Questionnaire (RCSQ); (b) anxiety levels measured using the Visual Analogue Scale-Anxiety (VAS-A); (c) stress levels via measuring the plasma melatonin, cortisone and norepinephrine; (d) levels of oxidative stress; (e) levels of inflammation; (f) the number of delirium-free days in 8 and 28 days; (g) the number of ventilator-free days in 28 days; (h) the number of antibiotic-free days in 8 and 28 days; (i) ICU length of stay; (j) overall ICU mortality at 28 days; (k) the incidence and severity of melatonin side effects in ICU patients.

### Study setting and population

The study setting is the comprehensive ICU, Fuxing Hospital, Capital Medical University, Beijing, China. All patients admitted to our ICU with acute respiratory failure requiring mechanical ventilation and tracheotomy to assist weaning are screened daily for study eligibility.

Inclusion criteria are: (a) age ≥18-years-old; (b) Glasgow Coma Scale (GCS) score ≥10; (c) sedation with propofol, morphine, alfentanil and dexmedetomidine has been discontinued for a minimum of 36 hours (48 hours for lorazepam and midazolam); (d) expected mechanical ventilation days ≥5 days; (e) patients are clinically and biologically stable and pass the weaning screening and are therefore ready to be weaned from mechanical ventilation.

Exclusion criteria are: (a) pregnant or breast-feeding women; (b) preadmission treatment of sleep disturbances; (c) a history of convulsions, psychiatric or neurological disease, sleep apnoea, deafness or blindness; (d) alcohol consumption ≥50 units per week or drug use; (e) liver insufficiency (child-Pugh class C); (f) renal insufficiency (requires dialysis); (g) severe heart failure (the New York Heart Association classification III/IV); (h) intestinal obstruction, ileus, gastroparesis or other conditions likely to affect the enteral absorption of melatonin; (i) the use of drugs that might alter melatonin secretion and decrease plasma levels of melatonin, such as benzodiazepines, NSAIDs, corticosteroids, beta-blockers, haloperidol and amiodarone [42, 43]; (j) known allergy to melatonin; (k) readmitted to the ICU after randomization to the study; (l) enrolled in another trial.

### Ethical aspects and informed consent

When a patient is identified as eligible for the study, immediate contact will be made with the 24-hour on-call study coordinator who will confirm eligibility. The attending physician will introduce the patient and their family to the study coordinator. The physician will make sure the patient and family know the credentials of the study coordinator and will explain that this person is going to discuss the study program that is being conducted and that this person is qualified to do so. Every relevant aspect of the project will be described. The study coordinator will stop frequently, ask if there are any questions, and request that the family repeat back in their own words what is being discussed to ensure understanding.

The study coordinator will explain that there is a possibility that the patient may suffer from poor sleep quality, and if so, the patient’s health could decline. The coordinator will say that there is a physiological sleep aid that provides good sleep quality without respiratory depression, while facilitating the patient’s condition. Additionally, he will explain that in a small percentage of patients, melatonin could cause headache, dizziness, nausea and drowsiness. The potential advantages of using or not using melatonin will be described. The study coordinator will be especially careful to assure the family that they are free to decline consent without consequences and that they can withdraw consent at any time without impact on treatment. Family members will be provided with contact information for the study coordinator, the local co-investigator and the local ethical committee. Written consent will be obtained in the presence of a witness. A register is kept of all patients evaluated for inclusion and of patients who withdraw from the study. The latter are clinically followed up without their data being analysed in the study.

The study protocol and consent forms were approved by the Institutional Review Board of Fuxing Hospital affiliated with Capital Medical University (approval number KY2014-22) and by the Chinese Clinical Trial Registry (approval number ChiCTR-TRC-14004319). The Fuxing Hospital’s Institutional Review Board gave positive advice for the addition of their ICU as the study site.

### Randomization with double-blind and allocation concealment

The study has a prospective, randomized, double-blind, placebo-controlled, parallel-group design. Eligible patients are randomly assigned to one of the two treatment study groups, labelled the ‘melatonin group’ or the ‘placebo group’. Randomization is based on a computer-generated random digits table and follows a concealed process using sealed and numbered envelopes that allocate the patient to either of the two arms of the study. Patients may be randomized into this study only once, unless they were discharged from the hospital and were re-admitted more than 180 days after their first enrolment. The study does not allow crossover, and if any occur, they will be reported as protocol violations.

The experimental drug and a placebo with the same characteristics are prepared by a pharmacist. The patients and all study personnel except the investigative pharmacist are blinded to the treatment assignment. The details of the treatment assignments are unknown to any of the investigators and are contained in a set of opaque and sealed envelopes, each marked with only the randomization number.

### Trial interventions

All patients are randomized at a ratio of 1:1 to the oral melatonin or placebo group. Time zero (T0) is defined by randomization and the study treatment and relevant inspections are started just after. The baseline data about the sleeping state of the patients needs to be recorded on day 1 after enrolment, thus the intervention begins on day 2. The melatonin group patients receive 3 mg of melatonin orally or through a feeding tube at 9:00 pm for four consecutive nights (from day 2 to 5) once they have enrolled in the study. If melatonin is administered by a nasogastric (or nasojejunal) tube, the tablet should be crushed and mixed with 20 ml of water, followed by another 20 ml to flush out the remnants inside the tube. The placebo group patients will receive the placebo orally or through a tube using identical methods and at the same time as the patients in the melatonin group.

After randomization, given the possible powerful effect of the high level of noise, turning on of the lights, or the intensive routine care and procedures on patients’ sleep in the ICU, we will offer earplugs and eye masks to the patients and we will dim the main light at night (from 9:00 pm to 7:00 am). Meanwhile, meetings and posters were employed to encourage staff to minimise environmental, nursing and clinical disturbances, such as clustering patient care activities during the nocturnal study periods. Additionally, nurses should select time periods to promote sleep by avoiding routine ICU care activities (such as the daily bath), reducing ambient noise and turning down the lights during these periods. Other therapy and nursing projects should follow the clinical guidelines and nursing criterion.

**Figure 1:**
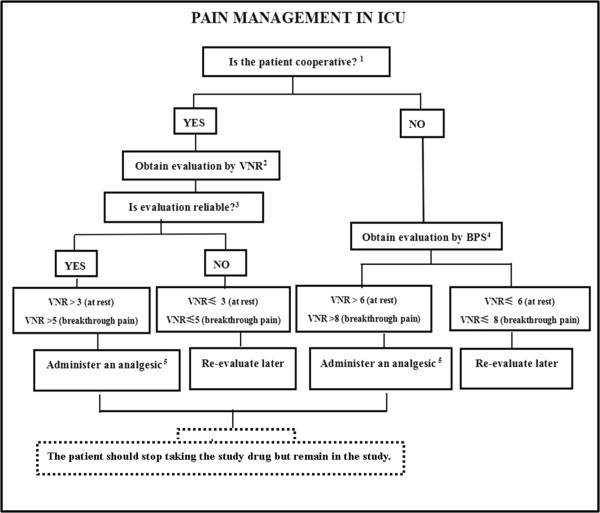
**A bedside flowchart for pain management in ICU patients.** ^1^Consider the patient not cooperative if: RASS < -2/CAM-ICU+/communication or linguistic barriers. ^2^Verbal Numeric Rating (VNR) 0 = no pain, 10 = maximal conceivable pain. Ask:“Can you quantify your pain between 0 and 10?”. Consider at rest and breakthrough pain (e.g. = coughing, tracheo-bronchial aspiration). ^3^Consider the evaluation as reliable if it takes into account the subjective parameters the patients uses to evaluate their pain: cultural, religious and familial aspects, expectation for secondary benefits. ^4^Behavioral Pain Scale (BPS) 0 = absence of pain, 12 = maximal pain. -Facial expression: 1. Relaxed/2. Partially tightened/3. Fully tightened/ 4. Grimacing. -Upper limbs: 1. No movement/2. Partially bent/3. Fully bent with finger flexion/4. Permanently retracted. -Compliance to ventilation: 1. Toleration movement/ 2. Coughing but tolerating ventilation for most of the time/ 3. Fighting ventilator/4. Unable to control ventilation.

**Figure 2:**
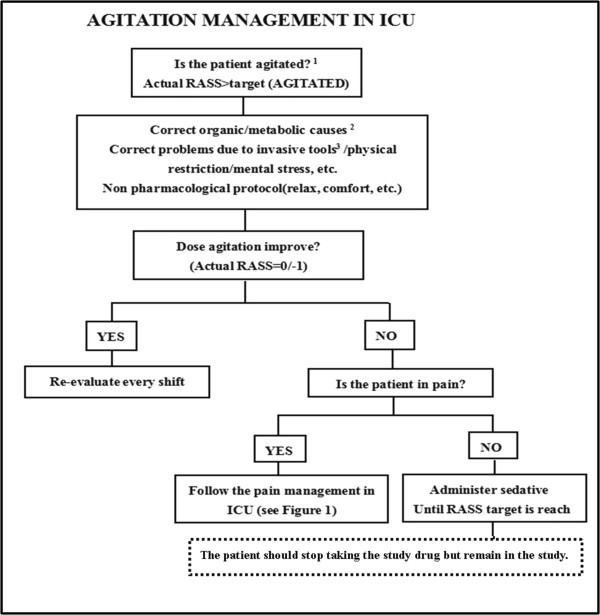
**A bedside flowchart for agitation management in ICU patients.** ^1^Always aim for RASS target =0/-1 (patient awake and tranquil, well adapted despite invasive tool and critical condition). RASS target may be between -2 to -4 if required by clinical conditions. ^2^Sepsis, hypo-perfusion, hypo/hyperglycemia, hypoxia, fever, electrolyte imbalance, alkalosis/acidosis. ^3^Mode of ventilation; bladder catheter positioning; bronchial aspiration.

**Figure 3:**
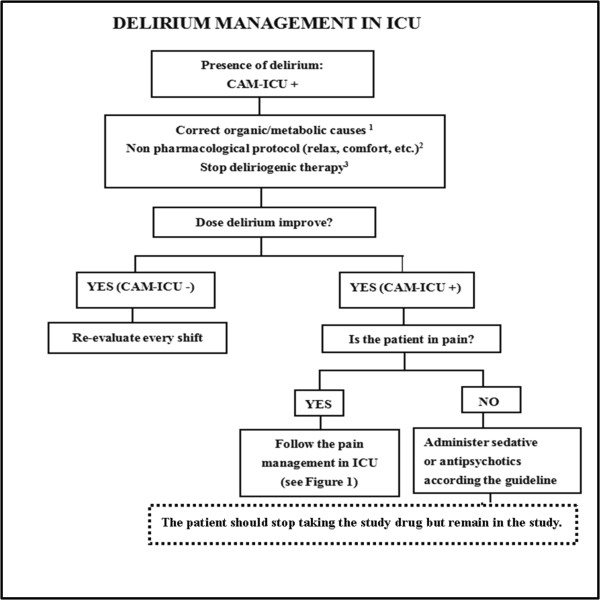
**A bedside flowchart for delirium management in ICU patients.** ^1^Sepsis, hypo-perfusion, hypo/hyperglycemia, hypoxia, fever, electrolyte imbalance, alkalosis/acidosis. ^2^None pharmacological protocol. Orientation: Use patient’s visual and auditory aids, Encourage communication calling the patient by name, Availability of patient’s personal belongings, Coherence between physicians and staff intervention, Use music or TV during the daytime. Environment: Lights off during the night, on during the daytime, Orient patients’ beds to allow vision of sunlight, Discourage sleep during the daytime, Patient mobilization and physiotherapy during the daytime, Control excessive noise during the daytime, Avoid medical and nursing procedures during the night. ^3^Consider to stop or decrease deliriogenic therapy: anticholinergic drug, metoclopramide, inhibitor of protonic pump, promethazine, etc.

**Figure 4:**
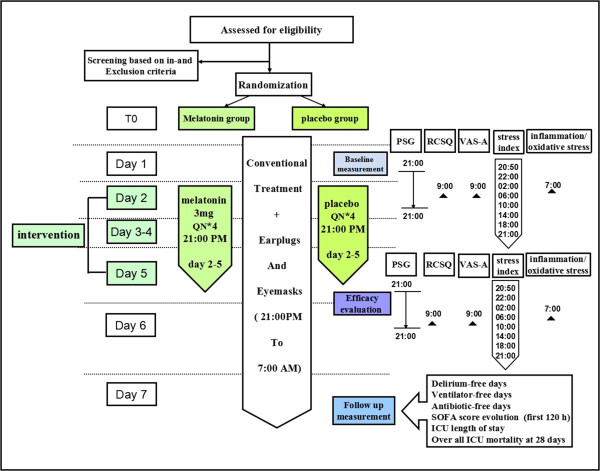
**The trial procedures flow sheet.** ICU, Intensive care unit; PSG, Polysomnography; RCSQ, Richards Campbell sleep questionnaire; SOFA, Sequential organ failure assessment; VAS-A, Visual analogue scale-anxiety.

In particular, during the sleep evaluation and intervention period (T0 time - 9:00 pm on day 6), pain, delirium and agitation will be managed in accordance with local guidelines. In case of clinical indication for analgesia and sedation, which can influence the effectiveness of melatonin and sleep structure, the study treatment is stopped and the necessary related administration is started according to the decision of the investigators. To help manage complex cases, three flowcharts are proposed for pain, sedation and delirium (Figures 1, 2 and 3). They stress the importance of considering all of those factors (that is, organic and metabolic causes, noise and light, anxiety, the presence of invasive tools and pain) that could contribute to agitation, delirium and pain, and should be corrected before administering any neuroactive drug. Analgesics (particularly opiates) must be used only in case of pain (a Verbal Numeric Rating (VNR) score >3 or a Behavioral Pain Scale (BPS) score >6) [44]. If delirium (CAM-ICU+) and agitation (RASS >0) appears, after correcting the underlying causes, the non-pharmacological protocol will be applied and potential deliriogenic therapies stopped; only after these interventions may physicians prescribe haloperidol or any other antipsychotic drug and remove these participants according to local guidelines. In these cases, the patient will stop taking the study drug but remain in the study and the investigator will perform the end-of-treatment assessments immediately and the end-of-study visit 28 days later. The trial procedures are shown in Figure 4.

### End of participation in the study

Patients will leave the study when the following occurs: (a) the patient refuses to participate in the study; (b) an exclusion criterion appears during the course of the intervention; (c) a severe adverse event occurs; (d) the patient’s condition gets worse; (e) ICU discharge or death.

### Data collection and management

Trained staff will record data and fill in the web-based collection forms. At the moment of patient inclusion, patient data on demographic characteristics and the history of past illnesses will be collected: age, sex, height and weight, Acute Physiology and Chronic Health Evaluation II(APACHE II) score, Sequential organ failure assessment(SOFA) score, Glasgow coma scale(GCS) score, diagnosis, and comorbidities. Clinical, physiological and analytical variables such as vital signs (temperature (T), blood pressure (BP), heart rate (HR), and respiratory rate (RR)), routine ICU laboratory tests (white blood count (WBC), serum creatinine (SCr), blood urea nitrogen (BUN), alanine aminotransferase (ALT), and aspartate aminotransferase (AST)) will be recorded at the moment of inclusion. The duration of time of ventilation, ventilation mode, the number of days in delirium, the ICU length of stay and the names, dose, duration of use and time of last use of sedatives, analgesics medications and vasopressor drugs will also be recorded prior to study inclusion.

Furthermore, sleep-related baseline data, including objective and subjective sleep quality and anxiety levels should be recorded. Subjective sleep quality, including the TST, the length of sleep at night (9:00 pm to 7:00 am) and in the day (7:00 to 9:00 pm), sleep architecture (NREM stage 1 and 2, SWS and REM), sleep disruption (the number of arousals and the duration of sleep), will be monitored for one 24-hour period using a portable PSG device from day 1 9:00 pm to day 2 9:00 pm. The PSG remains the gold standard for sleep quality assessment. Electroencephalographs (EEG; O1/M2, C4/M1), electromyographs (EMG), electrooculographs (EOG; right and left) and electrocardiographs (ECG; lead II) will be recorded. The patients’ skin will be prepared according to standard techniques. Gold cup EEG electrodes will be placed at O1/M2 and C4/M1 according to the International 10 to 20 System. Two EOG electrodes will be used for the right and left eye movements. The EMG electrodes will be located over the right and left masseter (facial) muscles. The electrode application will be performed by the specialist.

The subjective sleep quality refers to the subjective feelings of the nightly sleep status of the patients. The baseline nightly sleep perceptive quality (day 1 9:00 pm to day 2 7:00 am) will be evaluated using the RCSQ at 9:00 am on day 2. The RCSQ contains five 100 mm visual analogue scales (VAS): sleep depth, latency, awakenings, time awake and quality of sleep (higher scores indicate better sleep). The RCSQ was pilot tested in a medical ICU and validated with overnight PSG in medical ICU patients. In our study, patients who are unable to write were assisted; the patients used their current communication strategy to indicate where the investigator should mark the VAS.

Anxiety is defined as a state marked by apprehension, agitation, increased motor activity, arousal, and fearful withdrawal. The baseline anxiety levels are assessed on day 2 at 9:00 am via self-report using the 100 mm VAS-A, which is presented to patients with a vertical orientation such as a thermometer. The bottom of the scale is the statement ‘not anxious at all’ and the top is “most anxious ever.” Patients indicate their current level of anxiety in response to ‘How are you feeling today?’. The VAS-A score is the number of millimetres from the bottom edge of the line (not anxious at all) to the patient’s mark. The VAS-A and the Spielberger State Anxiety Inventory are correlated (r = 0.4916 to r = 0.8219), demonstrating concurrent validity.

During the intervention period (days 2 to 5), outcome-related data, such as the objective and subjective sleep quality, and the anxiety level should be recorded. Analyses are limited to the fourth day (day 5 at 9:00 pm to day 6 at 9:00 am) because the potential chronophypnotic benefits of melatonin are not immediate and may take at least three days to be realised. Therefore, objective sleep quality should be monitored for one 24-hour period using the PSG from day 5 at 9:00 pm to day 6 at 9:00 pm, and the associated parameters and methods as described in the ‘Methods/Design’ section. Meanwhile, the subjective sleep quality and anxiety levels should be evaluated at 9:00 am on day 6 and the related methods have been previously described. In addition, daily information including the vital signs and lab parameters, the SOFA score, neurological monitoring (RASS, CAM-ICU, and GCS), as well as the mode, parameter and duration of mechanical ventilation should be recorded.

The sound and illuminance levels are recorded from day 1 to day 6 using the integrated sound pressure level meter and the illuminance level meter. Continuous equivalent sound pressure levels (Leq) in ‘A’ weighted decibels and peak sound pressure levels (Lpeak) in ‘C’ weighted decibels are logged every second. The illuminance level (in lux) is recorded once per minute using a sensor placed close to the patient’s head. The bedside nurse should be asked to log an event whenever the patient received treatment or care. The event log will contain the following items: clinical assessment, tracheal suctioning, pressure area care, physiotherapy, mouth or eye care, blood test (sampling), washing, non-invasive blood pressure, eating and drinking, dressing, pain, line insertion and X-ray. Drug records are compiled daily for drugs that are known to adversely affect sleep or melatonin pharmacokinetics.

At the time of the ICU discharge, patients’ data and vital statuses, lengths of mechanical ventilation, the number of days of antibiotics use, the number of delirium-free days, the length of the ICU stay and ICU mortality will be recorded.

To investigate the enteral absorption and metabolism of orally administered melatonin in critically ill patients during their ICU stay and to evaluate the serum melatonin levels and their circadian variations in these patients, the melatonin levels are measured in blood samples taken on days 1 and 2 (before the intervention) and days 5 and 6 (four days after the intervention) at 20:45 (before administration), 9:10 pm, 9:30 pm, 00:00 am, 3:00 am, 6:00 am, 2:00 pm and 8:00 pm in 20 patients from each group. The plasma levels of norepinephrine and cortisol are also measured at the same time points. In addition, plasma levels of SOD, MDA, catalase (CAT), IL-6 and IL-8 are measured at 07:00 am on days 2 and 6.

### Current sample size justification

Primarily, we expect an increase in the time of the patient’s night sleep after melatonin administration in ICU patients with sleep deprivation. Based on retrospective data from our institution in the same population (mean TST from the nightly sleep was 3.5 hours with a SD of 1.8 hours). A 20% (0.7 hour) difference in the nightly sleep time between the two study arms is considered clinically relevant and likely to influence practice. Using the Power Analysis and Sample Size for Windows software (PASS2000, NCSS, Kaysville, Utah, United States), we will need to study 82 experimental subjects and 82 control subjects to be able to reject the null hypothesis that the population means of the experimental and control groups are equal with a probability (power) of 0.8 by the two-side paired *t*-test. The Type I error probability with testing this null hypothesis is 0.05. Assuming a dropout rate of 20%, 99 patients per group will be recruited.

### Statistical analysis

All analyses will be performed according to the intention-to-treat principle, that is, all randomized patients will be analysed in the groups to which they were originally allocated and will be blinded to treatment assignment. Baseline patient characteristics will be compared and described using the appropriate statistics. Categorical variables will be presented as the numbers and percentages and will be analyzed by the *χ*^2^- tests or Fisher’s exact tests, or when appropriate, as relative risks. Continuous variables will be checked for normal distribution by the Kolmogorov-Smirnov test. Normally distributed variables will be expressed by their mean and standard deviation; non-normally distributed variables will be expressed by their medians and 95% confidence levels. Comparisons of continuous variables will be performed using Student’s *t*-test for normally distributed variables and the Mann-Whitney U test for non-normally distributed variables. Appropriate (linear, logistic, or Poisson) regression will be generated for the identification of the determinants of outcomes and for the correction of baseline covariates.

One-way repeated measures analysis of variance (ANOVA) were used to determine differences in the sleep variables, the subjective sleep quality, anxiety levels and plasma levels of melatonin, cortisol and norepinephrine. Twenty-eight day mortality will be analysed according to the Kaplan-Meier method and the results will be compared with the log-rank test hazard ratios and reported with 95% confidence limits. The number of delirium-free days, ventilation-free days and antibiotics-free days will be tested by Student’s *t*-test for normally distributed data and the Mann-Whitney U-test for non-normally distributed data. The differences in adverse events between the intervention and placebo groups are used for safety analyses. All tests of significance will be at the 5% significance level and two-sided. All of the data in this trial will be assessed with SPSS Statistics version 20.0 (SPSS, Chicago, Illinois, United States). The main analysis will be fully specified before unmasking.

### Dissemination policy

According to the Standard Protocol Items: Recommendations for Interventional Trials (SPIRIT) guidelines, the authors declare that data that break the blinding will not be presented prior to the release of the mainline results. Breaking of the blinding will occur at the end of the study. A clinical article will be written on the primary (and secondary) outcomes of the study and the results will be disseminated regardless of the magnitude or the direction of effect. The present trial is not industry-initiated. As such, there are no publication restrictions imposed by sponsors.

In addition, a full study report, an anonymized participant-level dataset and the statistical code for generating the results will be made publicly available no later than three years after the termination of the study for sharing purposes.

## Discussion

This study is the first large sample size, prospective double-blind, ramdomized, placebo-controlled trial powered to test the hypothesis whether melatonin improve the sleep quality in ICU patients. Previous researches mainly had small sample size, and they did not use the PSG, the gold standard for assessing sleep, to evaluate sleep quality and ignored the importance of monitoring the all-day sleep. Therefore, our study is also the first to use 24-hour PSG as monitoring tools to evaluate the effect of melatonin on sleep quality in ICU patient. However, the authors acknowledge the limitation of only including patients who pass the weaning screening and are therefore ready to be weaned from mechanical ventilation, and not including who are administered medication for sedation and analgesia. Therefore, the enrolled patients might be inclined to have the longer length of ICU stay or be relative milder case. So we can not investigate most of ICU patients who are just admitted to ICU or treated intubation and mechanical ventilation.

Even so, significant differences in the safety and/or efficacy endpoints will provide important evidence for improving ICU patient sleep quality. A neutral result will also provide important insight, as this would mean that more studies are needed to evaluate the safety and efficacy of melatonin for ICU sleep deprivation.

## Trial status

The study has been initiated as planned in February 2014. One interim analysis advised continuation of the trial. The study will be completed in February 2015.

## Abbreviations

APACHE II: Acute Physiology and Chronic Health Evaluation II score
BP: Blood pressure
BPS: Behavioural pain scale
CAM-ICU: The confusion assessment method for the intensive care unit
CAT: Catalase
COPD: Chronic obstructive pulmonary disease
ECG: Electrocardiograph
EEG: Electroencephalograph
EMG: Electromyograph
EOG: Electrooculograph
GCS: Glasgow coma scale
HR: Heart rate
ICU: Intensive care unit
IL: Interleukin
MDA: Malonaldehyde
NREM: None rapid eye movement
NSAIDs: Non-steroidal anti-inflammatory drugs
PSG: Polysomnography
RASS: Richmond agitation sedation scale
RCSQ: Richards Campbell sleep questionnaire
REM: Rapid eye movement
RR: Respiratory rate
SCN: Suprachiasmatic nuclei
SOD: Superoxide dismutase
SOFA: Sequential organ failure assessment
SpO2: Peripheral oxygen saturation
TST: Total sleep time
SWS: Slow wave sleep
VAS-A: Visual analogue scale-anxiety
VNR: Verbal numeric rating.
